# Implementing an Internal Audit: Evaluating Hand Scrub Compliance in a Tertiary Care Hospital

**DOI:** 10.7759/cureus.64778

**Published:** 2024-07-17

**Authors:** Ahmed Mohamed Yousif Mohamed, Abubakr Muhammed, Faris Jamalaldeen Mohammed Hamed, Amir Malik Ibrahim Algak, Elamin Ezeldin Abdelrhim Attaelmanan, Ahmed Sufyan Ahmed Abdalla, Mohammed AlSiddig Modawy Alkheder, Mustafa Sabir Abakar Awad

**Affiliations:** 1 Orthopaedics and Trauma, Burjeel Medical City, Abu Dhabi, ARE; 2 General Surgery, University of Gezira, Wad Madani, SDN; 3 General Surgery, Al Neelain University, Khartoum, SDN; 4 General Surgery, Omdurman Islamic University, Omdurman, SDN; 5 Microbiology, Al Neelain University, Khartoum, SDN

**Keywords:** world health organization, asepsis, surgical site infection, clinical audit, hand scrub

## Abstract

Background

Aseptic protocol adherence and sterilization are the most important factors in a patient's satisfactory recovery after surgery. The standard hand scrubbing procedure helps control infection and keeps the surgical site clean by adhering to aseptic principles.

Methods

Thirty-six young residents and house officers participated in this prospective audit after ethical clearance was obtained. The World Health Organization (WHO) standard criteria were adhered to both before and after the intervention. Participants were observed in the surgical operation theatre (OT) without prior notice to ensure hand hygiene compliance before surgical procedures. The intervention included a video presentation as well as a live demonstration.

Results

Only 64.41% (n=23) of residents and house officers followed the recommended standard hand hygiene procedures before the intervention. This percentage rose to 93.92% (n=33) following the intervention, suggesting a noteworthy improvement.

Conclusion

Significant changes in the acceptance rates for the essential requirements of hand hygiene were observed after the evaluation in the second cycle. Adhering to WHO guidelines for procedures will help reduce the risk of infections and promote awareness of asepsis in practice.

## Introduction

Surgical site infection (SSI) is one of the most common types of hospital-acquired infections (HAIs) in developing countries [1], and it is one of the most significant nosocomial infections that occur in surgical departments, increasing the risk of morbidity and mortality as well as adding to expenses [2]. SSIs are defined as infections that affect the incision or deep tissue at the operation site and manifest within 30 days following surgery or within a year if an implant is left in place following the procedure [3]. Infections impact 5-10% of hospitalized patients in developed nations, making them the most common side effect after a stay [4]. Though it's a fairly basic habit, only about 40% of healthcare professionals wash their hands frequently. The substitution of waterless alcohol-based products for traditional handwashing has been a major change recommended for hand hygiene practices [5].

The frequency of contamination decreases when alcohol-based handwash with ethanol and isopropyl alcohol replaces non-medicated chemicals because the microorganisms on the operator's hands are destroyed. Assessing HAIs and reporting them effectively is essential for identifying control mechanisms in healthcare systems and putting the required changes into place. However, such monitoring can be expensive, which presents a major challenge to global healthcare systems, especially in developing nations [6].

We believe that the rushed environment of the operating room (OR) in Sudan, the short training duration, and the dearth of highly qualified medical staff make it difficult for general surgery residents and house officers to acquire this skill. Before starting any surgical procedure, we should follow World Health Organization (WHO) guidelines regarding hand hygiene [7]. The WHO states that rather than concentrating only on the kind of hand hygiene products, healthcare workers should also be encouraged to practice good hand hygiene by focusing on specific elements that are currently known to have a major impact on behavior. This surgical audit aimed to assess the residents' and house officers' handwashing practices in the General Surgery and Orthopaedic Department.

## Materials and methods

This audit was conducted in the General Surgery and Orthopaedic Department of a tertiary hospital in Sudan, involving 36 participants from surgical departments, including residents and house officers, in November 2023. Approval was obtained from the Institutional Ethics Committee (IEC) of Al Manaqil Teaching Hospital (approval number: SUD23/11/MAN-22).

Participants were observed over a two-week period as they cleaned their hands before undergoing surgical procedures. It should be noted that they were not aware that they were being observed or that their hand-cleansing practices were being audited. The observation was compared with the WHO's handwashing recommendations. The procedure outlined in Table 1 assesses the participants' handwashing skills. Out of a total of 11 steps for hand scrubbing, each "yes" in Table 1 indicates a step that was done correctly, and each "no" indicates a step that was done incorrectly. We compiled the results for each of the 11 hand hygiene criteria after assessing the adherence of each participant to them. These percentages were primarily used to assess the degree to which each participant generally complied with the criteria.

**Table 1:** Pre-evaluation and post-evaluation compliance percentage improvement for various steps in hand scrubbing %: percentage; N: number

Items of scrub	Adherence rates				% improvement
	First cycle		Second cycle		
	%	N	%	N	
Wet the hands and forearms	100	36	100	36	-
Rub palm to palm with fingers interlaced	75	27	94.45	34	19.45
Scrub the right palm over the back of the left hand and vice versa with fingers interlaced	63.89	23	77.80	28	13.91
Rotational rubbing backwards and forwards with the clasped finger of the right hand into the left hand and vice versa	41.67	15	83.34	30	41.67
Rotational rubbing of the right thumb clasped in the left hand and vice versa	52.78	19	94.45	34	41.67
Rub fingertips on palms for both hands	36.11	13	100	36	63.89
Continue with rotational action down opposing arms working to the elbow for one minute	69.45	25	97.25	35	27.80
Rinse hands and arms by passing them through the water in one direction, only from fingertips to elbow	75	27	100	36	25
Do not move the arms back and forth through the water	36.11	13	86.12	31	50.01
Do not splash the water onto the dress	75	27	100	36	25
Hold the hands above the elbow at all times	80.60	29	100	36	19.40
Mean compliance	64.14	23	93.94	33	29.80

After the initial two weeks of observation and data collection, each participant received a presentation that included a live demonstration and a video illustrating proper handwashing according to WHO guidelines. Additionally, we placed an illustrated handwashing instruction manual in the operating room.

In the second phase of the audit, all participants were reobserved for compliance with hand scrubbing over a period of two weeks. For hand cleaning, all 11 stages were awarded one mark for each step that was completed correctly. Each participant received a total score, and the average improvement was calculated by adding together all of the compliance scores. For each participant, we determined compliance with these 11 criteria for handwashing. We then added each participant's scores to each criterion separately. Percentages were used to determine how well each participant overall conformed to each criterion.

## Results

Thirty-six surgical participants were randomly assigned to a cohort, and their handwashing skills were closely observed. Initially, 64.14% (n=23) of participants followed the recommended hand hygiene guidelines during pre-sensitized observation. There was a noticeable improvement following thorough training that included a thorough video demonstration and live demonstration. The effectiveness of the interventions was demonstrated by the notable increase in the success rate to 93.94% (n=33) (Table 1). Each criterion saw significant increases in adherence rates, leading to a higher overall mean compliance rate post-evaluation.

Table 1 showed that the criterion "scrubbing palms with digits" had the greatest improvement, with a 63.89% (n=23) improvement. This enhancement highlights how real-world examples can improve participants' compliance with appropriate handwashing practices. Specifically, "rubbing fingertips on palms for both hands" is a critical step recommended in guidelines for effective hand hygiene. This technique ensures thorough cleaning or sanitization of fingertips, crucial for reducing the transmission of infections.

**Figure 1:**
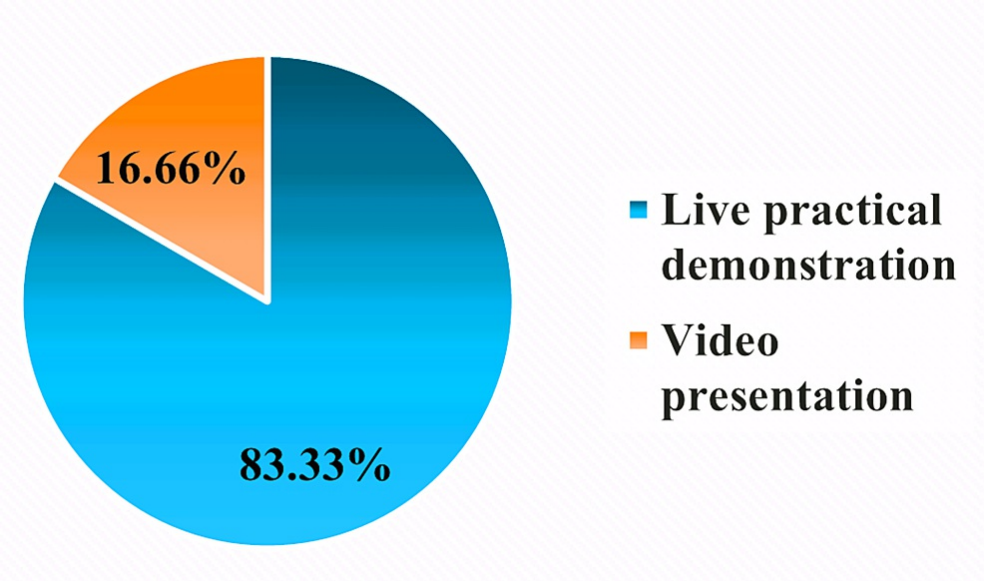
Best intervention method according to participants

We also inquired with the participants about the most effective intervention that enabled them to understand the standard hand scrubbing technique. As illustrated in Figure 1, 83.33% (n=30) of participants chose live, practical demonstration over video presentation as the most effective way to teach the technique.

## Discussion

Early in the 19th century, handwashing procedures were introduced into patient care settings. When combined with other hand hygiene measures, the practice has proven to be extremely important over time and has reduced the pathogens that cause HAIs and nosocomial infections [8-10]. Healthcare professionals' contaminated hands are the main way that pathogens spread. Therefore, maintaining good hand hygiene reduces the growth of microorganisms, which lowers the risk of infection and lowers overall medical expenses, hospital stays, and, eventually, reimbursement. Hand hygiene is the single most important practice in reducing the transmission of infection in the healthcare setting, according to the Centers for Disease Control and Prevention (CDC) [11]. In spite of this evidence, research has consistently demonstrated that healthcare professionals do not sufficiently understand the value of hygiene and compliance rates are still low [12].

A distinct set of skills is needed for surgical hand antisepsis or hand hygiene before surgery compared to standard handwashing [13]. SSIs are among the most frequent types of hospital-associated infections for surgical patients, and they can arise from the unintentional transfer of microorganisms to a patient's surgical site [14]. Therefore, practicing good hand hygiene before surgery can help lower the risk of SSIs.

As per 57 studies carried out by diverse experts worldwide, there was an improvement in hand hygiene compliance following the intervention [6], and according to a study conducted by Mukherjee et al. (2021), 33.59% (n=14) of 42 undergraduate general surgical internees initially met the baseline compliance level, with 100% compliance achieved after six tries [15]. 

After reviewing every study that had been done and the current survey that our study had conducted, we concluded that, in order to increase compliance among healthcare workers and transfer knowledge, it was necessary to regularly re-audit and sensitize pictorial guides, video demonstrations, and individual demonstrations.

We are aware of this study's potential limitations. The audit was conducted at a single center, and the small sample size could affect the generalizability of the results. Additionally, reliance on direct observation may introduce bias, as participants might alter their behavior when being observed. The short duration of the study and the lack of a control group further limit our ability to attribute improvements solely to the audit intervention. Variations in individual compliance, due to differences in background, experience, and personal habits, were not accounted for and could impact the outcomes. External factors such as workload, resource availability, and institutional support, which were not controlled, might also influence adherence rates. Despite these limitations, the notable improvements observed suggest a positive trend towards better hand hygiene practices, warranting larger, multi-center studies to confirm these findings and explore long-term sustainability.

## Conclusions

This clinical audit underscores the importance of ongoing assessments and training to enhance patient safety. It also emphasizes the importance of hand scrubbing to reduce infection rates resulting from HAIs. The effectiveness of training in improving adherence to WHO-recommended hand scrubbing procedures is evident from significant post-intervention improvements. This progress underscores the potential of regular evaluations and continuous education to promote better compliance with hand hygiene and surgical protocols.
